# A Child-Centered Framework for Determining Mental Distress Severity and Liability: Evidence from Chinese Judicial Practice

**DOI:** 10.3390/bs16030388

**Published:** 2026-03-08

**Authors:** Qidi Xue, Dongqing Yu, Zexin Zhang

**Affiliations:** 1 Faculty of Education, Northeast Normal University, Changchun 130024, China; xueqd619@nenu.edu.cn (Q.X.); yudq049@nenu.edu.cn (D.Y.); 2 School of Law, Jilin University, Changchun 130012, China

**Keywords:** preschool children, mental distress, best interests of the child, child participation, capabilities approach, Lundy model, China

## Abstract

Compensation for mental distress in preschool children is a crucial mechanism for protecting their personality rights, yet current judicial practice in China relies heavily on judicial discretion and lacks child-sensitive standards for determining severity. Following the enactment of the Preschool Education Law of the People’s Republic of China in 2025, the principle of the Best Interests of the Child has placed new behavioral and developmental requirements on decision-making, particularly regarding the recognition of children’s expressive limitations and psychological vulnerability. Drawing on representative judicial cases, this research identifies inconsistencies in current adjudication—primarily between factual presumption and medical proof—and highlights their failure to reflect preschoolers’ developmental characteristics. To address this gap, we construct a child-centered liability determination framework integrating the Lundy model of child participation and Nussbaum’s Capabilities Approach. This framework provides a structured method for incorporating children’s voices into proceedings and offers multidimensional criteria for assessing capability impairment as an indicator of mental distress severity. These findings suggest that the framework can help reduce excessive discretion, strengthen developmental sensitivity, and promote more consistent and equitable adjudication. Beyond the Chinese context, this research offers an analytical lens for advancing international discussions on child-centered mental distress assessment and children’s rights protection.

## 1. Introduction

Mental distress, as a significant form of personality rights infringement, has gained increasing global recognition in recent years. Personality rights are the rights of persons governed by civil law, such as the right to life, the right to corporeal integrity, the right to health, the right to name, the right to entity name, the right to likeness, the right to reputation, the right to honor, the right to privacy, etc., as stipulated in Article 990(1) of the Civil Code of the People’s Republic of China. Infringement of personality rights refers to situations where personality rights are violated. Compensation for mental distress is typically applicable when personality rights have been severely violated (Kasperska, 2021). The role of mental distress compensation extends beyond compensation and consolation to include functions such as punishment of wrongful acts and maintenance of social order (Qu & Wang, 2020). In most jurisdictions, compensation is primarily monetary and remains the principal legal remedy (Lewinsohn-Zamir, 2013). Thus, although compensation for mental distress cannot fully eradicate psychological suffering, it can provide a measure of solace and rights vindication for the victim (Liu, 2024).

In Chinese judicial practice, compensation is generally conditioned upon proof of “severe” mental distress. This requirement aims to prevent excessive claims and reflects the principle *de minimis non curat lex* (X. Yang, 2025). However, preschoolers, as highly vulnerable rights-holders, are often assessed under adult-oriented severity standards. Such standards insufficiently account for children’s developmental characteristics and modes of psychological expression. This institutional bias undermines the substantive protection of preschoolers’ personality rights. The Preschool Education Law, effective 1 June 2025, introduces the “Best Interests of the Child” principle into the educational legal framework, establishing a normative foundation for safeguarding child welfare in civil law. Yet, its operationalization within mental distress compensation remains unclear, particularly regarding evidentiary participation and the assessment of severity.

Addressing this gap requires theoretical tools capable of translating child-rights principles into adjudicative practice. Article 12 of the United Nations Convention on the Rights of the Child establishes children’s right to express their views and to have those views given due weight in decision-making. To operationalize this legal principle within institutional contexts, the Lundy Model of Participation was developed by Laura Lundy as a conceptual framework grounded in international children’s rights law (Lundy, 2007). The model conceptualizes meaningful participation through four interrelated components. The first is space, which ensures safe and inclusive opportunities for children to express their views (Kennan et al., 2021). The second is voice, which requires that children receive adequate information, support, and accessible means of communication to form and articulate their opinions (Correia et al., 2022). The third is audience, emphasizing that children’s views must be heard directly by decision-makers (Molloy, 2024). The fourth is influence, which ensures that children’s views shape outcomes and that feedback is provided (Molloy, 2022). These dimensions operate as an integrated participatory process, and the absence of any element undermines the realization of participation rights (Murray et al., 2020). The model has been adopted at policy and institutional levels by international organizations and governmental bodies. For example, institutions such as the European Commission and UNICEF have incorporated the model into professional training and child-participation initiatives (Kennan et al., 2018; Vilgeus Loy & Kneck, 2025). Such cross-sectoral and international uptake demonstrates not only the model’s practical applicability but also its normative credibility and institutional reliability (Lundy et al., 2011). Its conceptual clarity and operational structure make it a robust framework for evaluating the extent and effectiveness of child participation in judicial decision-making contexts.

While the Lundy model addresses participatory processes, the assessment of severity requires an evaluative framework grounded in children’s developmental realities. The Capabilities Approach, initially proposed by Amartya Sen and further elaborated by Martha Nussbaum, provides such a perspective (Schweiger, 2025). The approach evaluates the substantive freedoms that individuals must be afforded to achieve their functional capabilities. Its analytical focus is on what individuals are effectively able to be and to do, and it highlights the challenge of addressing human diversity under conditions of persistent inequality and injustice (MacKenzie et al., 2023). Nussbaum advances a more normative account by proposing a list of ten central capabilities that define the minimum requirements for a life with dignity. These include, for instance, life, bodily health, bodily integrity, imagination and thought, emotions, practical reason, affiliation, relationships with other species, play, and control over one’s environment (Thomson, 2021). Nussbaum argues that capabilities are interdependent and non-fungible, and impairment of one weakens others. Accordingly, the seriousness of mental distress depends on whether it substantially restricts the ability to exercise these core capabilities. While minor distress may fall below this threshold, severe psychological harm undermines human development and dignity, justifying heightened compensation (Nussbaum, 2006). Moreover, Nussbaum’s framework aligns with international human rights law. The capabilities of life and bodily integrity correspond to Article 4 of the UNCRC and Article 10 of the United Nations Convention on the Rights of Persons with Disabilities. The framework also aligns with international human rights law, reinforcing its legitimacy as a standard for evaluating non-pecuniary harm (Vecchio & Martens, 2021).

Despite the growing recognition of the Best Interests of the Child principle, its operationalization within mental distress compensation remains limited. Existing adjudication frameworks rely heavily on adult-oriented evidentiary standards and lack structured mechanisms for incorporating children’s voices or assessing developmental harm. This gap highlights the need for an analytical model capable of translating child-rights norms into judicially applicable criteria. To address this limitation, this research draws on typical cases of mental distress compensation involving preschoolers in China. It integrates the participatory dimensions of the Lundy model with the evaluative framework of the Capabilities Approach. Through this theoretical synthesis, this research constructs a child-centered framework for determining severity in compensation claims. Guided by this rationale, this study seeks to answer the following question: How can a child-centered participatory and capability-based framework improve the judicial determination of mental distress severity in compensation cases involving preschoolers? By addressing this question, the study evaluates the framework’s judicial applicability, examines its capacity to resolve adjudicative dilemmas, and proposes pathways to refine China’s mental distress compensation mechanisms in line with the Best Interests of the Child principle.

## 2. Literature Review

### 2.1. The Best Interests of the Child and Child Participation

The *United Nations Convention on the Rights of the Child* (UNCRC) is the most widely ratified human rights treaty (Waldock, 2016). It reflects the international community’s commitment to protecting preschoolers from violence, exploitation, and discrimination. It also affirms that human rights apply to all preschoolers, regardless of political, social, or cultural context (Berrick et al., 2022). The UNCRC establishes four fundamental principles: the principle of non-discrimination (Article 2), the right to survival and development (Article 6), the opportunity to participate in decision making (Article 12), and most importantly, the Best Interests of the Child principle (Article 3). Article 3 explicitly states: “In all actions concerning children, whether undertaken by public or private social welfare institutions, courts of law, administrative authorities or legislative bodies, the best interests of the child shall be a primary consideration” (Sutherland & Macfarlane, 2016). Since China ratified the UNCRC in 1991, it has gradually incorporated the Best Interests of the Child principle into domestic law, most notably through the Preschool Education Law, which elevates the principle to a basic legal norm (Gresdahl et al., 2025). Comparative jurisprudence, such as French constitutional case law and the Inter-American Court of Human Rights’ interpretation of Article 19 of the American Convention, demonstrates the universal applicability of the Best Interests of the Child principle (Fuentes & Vannelli, 2021).

While the Best Interests of the Child principle has become the cornerstone of child rights protection worldwide, it does not operate in isolation. Importantly, the UNCRC’s recognition of children as independent rights-holders establishes a conceptual link between the best interests principle and Article 12. Article 12 states that children capable of forming their own views have the right to freely express those views in all matters affecting them and to have those views given due weight in accordance with their age and maturity (Hu, 2020). China has articulated children’s participation rights in the National Human Rights Action Plan (2016–2020) and related policy documents. These frameworks encourage children to express their views across institutional settings and expand channels for their participation (X. Wang et al., 2024). Xiao and Li (2024) argue that child participation in civil proceedings operationalizes the principle of the Best Interests of the Child. They argue that without genuine opportunities for children to be heard in decision-making processes, the principal risks remain procedurally symbolic. Participation, therefore, functions both as an independent right and as a necessary condition for the realization of the best interests of the child.

Beyond its international law obligations, China has progressively embedded the Best Interests of the Child principle within its domestic legal order (D. Wang & Wang, 2021). The Law of the People’s Republic of China on the Protection of Minors, as the cornerstone of the country’s child protection framework, explicitly enshrines in Article 4 the “principle of the best interests of the child” as a fundamental norm governing the protection of minors. This legislative mandate is further reinforced by constitutional foundations: Article 49 of the Constitution of the People’s Republic of China establishes the state’s protective duty by stipulating that “marriage, the family, and mother and child are protected by the state”, thereby providing constitutional grounding for child welfare as a matter of public concern.

Importantly, the normative integration of the Best Interests of the Child principle extends to the recognition of children’s participatory rights. Article 3 of the Law on the Protection of Minors explicitly guarantees children’s rights to “participation”, while Article 19 imposes a legal obligation on parents or guardians to heed children’s opinions in decisions affecting them. This domestic legal architecture aligns with and operationalizes Article 12 of the UNCRC, transforming children’s right to be heard from an abstract international standard into a judicially cognizable domestic requirement. Overall, incorporating these principles into law as general guidelines and granting corresponding rights to protection and participation signifies a degree of state recognition of children’s autonomy (Biddulph & Rosenzweig, 2019). China has further articulated these commitments in its national policy frameworks, including the National Human Rights Action Plan, and has explicitly linked its child protection efforts to the United Nations Sustainable Development Goals (SDGs), particularly Target 16.2 on ending violence against children, as reflected in its periodic reporting with regard to UN human rights mechanisms. This multi-layered normative framework, spanning constitutional principles, statutory mandates, policy directives, and international commitments, collectively establishes the legal foundation for child-centered adjudication in matters affecting preschoolers’ welfare.

### 2.2. Compensation for Mental Distress and Severity

Restricting compensation for mental distress by establishing specific conditions for its application is a common measure adopted in the legislation of various countries, with the most representative requirement being that the mental distress must reach a high severity level (Feng & Ran, 2025). A global review of legal provisions on compensation for mental distress reveals significant differences in how jurisdictions assess the severity of distress. For example, Article 195(1) of the Civil Code of China’s Taiwan region is an example of provision emphasizing that mental distress must reach a severe extent. Conversely, the laws of some countries or regions do not have a severity threshold for compensation regarding mental distress. Section 253 of the German Civil Code (BGB) adheres to the principle that damages warrant reasonable compensation, meaning compensation for mental distress may be awarded without “severity”. The Civil Codes of France and Switzerland, among other jurisdictions, share the same standard as Germany. Currently, the assessment of mental damages for young children in China is conducted by judges in judicial cases, with the specific criterion being the severity standard. Regarding the institutional choice for compensation for mental distress, both legal provisions and judicial practice in China indicate that the tortfeasor bears the liability for mental distress only when they cause outcomes characterized by “severity”. The Supreme People’s Court of the People’s Republic of China first proposed the concept of “compensation for mental distress” in the 1993 Reply of the Supreme People’s Court to Several Questions Concerning the Trial of Cases Involving the Right of Reputation. The 2001 Interpretation of the Supreme People’s Court on Several Issues Concerning the Determination of Compensation Liability for Mental Distress in Civil Torts systematized the framework for mental distress compensation, expanding its scope to include not only traditional personal rights but also personal dignity and memorial items of symbolic significance. The Civil Code of the People’s Republic of China, which came into effect on 1 January 2021, addresses mental distress compensation in Article 583 of the Contract Book, Article 996 of the Personality Rights Book, and Article 1183 of the Tort Book, thereby forming a distinctive “three-source parallel” system (Hong, 2024). However, despite the establishment of this general framework, there remains no clear and unified standard for determining liability for mental distress compensation (Luo, 2024). The concept of severity as an essential element is not a strict legal concept; it possesses a degree of uncertainty in both its semantic scope and normative purpose (Zhu, 2022). Although the Interpretation was revised and adopted again on 23 December 2020, it continues to lack unified criteria, particularly regarding children. This institutional limitation reflects a broader and persistent shortcoming: the insufficient recognition of preschoolers’ special status as rights-holders (Byrne & Lundy, 2019).

Chinese judicial practice adopts the requirement of severe damage for mental distress compensation, yet its necessity remains controversial in academia. Some views hold that the severity of the damage consequence can generally only be a factor considered in determining the amount of compensation for mental distress, not a threshold for bearing liability (Zhang, 2009). Other views argue that allowing compensation regardless of the magnitude of the damage may lead to frivolous litigation, overburdening the courts while distorting the regulatory mechanism of interpersonal relationships in a healthy society (Xie, 2010). Overall, existing scholarship on mental distress compensation in Chinese civil law focuses mainly on general theory, with few studies focusing on the principle of “the best interests of the child.” However, the exercising of both children’s participation rights and their procedural participation rights can have a substantive impact on matters affecting the vital interests of minors (Keddell, 2023). As a result, compensation for mental distress in preschoolers remains under-studied, reducing its practical relevance. This research addresses this gap by examining the relationship between the Best Interests of the Child principle and compensation for preschoolers’ mental distress. Aligning with Chinese judicial practice, it reinterprets the “severity” requirement through the lens of the child’s best interests.

## 3. Methods

### 3.1. Research Design

This research adopts a qualitative multiple-case-study design to investigate how Chinese courts determine the severity threshold in compensation claims for preschoolers’ mental distress. The purpose is not statistical generalization but doctrinal and theoretical insight (Yin, 2018). The multiple-case approach supports both within-case analysis and cross-case comparison, enabling the identification of adjudicative patterns, doctrinal inconsistencies, and gaps in the consideration of children’s developmental particularities (Stake, 2013). Case selection followed a purposive sampling strategy (Tajik et al., 2025). Rather than aiming at representativeness in a statistical sense, purposive sampling enables the selection of cases that are information-rich and theoretically relevant to the research question (Nyimbili & Nyimbili, 2024). In particular, typical case sampling involves selecting typical, representative cases within the research phenomenon, enabling the identification of adjudicative patterns, doctrinal inconsistencies, and gaps in the consideration of children’s developmental particularities (Campbell et al., 2020). This study selected seven typical litigation cases concluded between 2017 and 2024 as the analytical sample. The specific criteria for case selection were as follows:

1. Subject Criteria: The parties involved in the cases included preschool children (typically referring to ages 3–6), and the plaintiffs were the child or their legal representative.

2. Cause of Action Relevance: The cases involved the liability of kindergarten education institutions. This specifically encompassed typical scenarios such as the unilateral dismissal of a child by the kindergarten, accidental injuries sustained by the child within the kindergarten, and instances of verbal abuse or physical punishment of the child by teachers.

3. Time Frame: Cases concluded between 2017 and 2024 were selected to reflect the latest developments in judicial practice in the context of the enactment of the Civil Code and the formulation of the Preschool Education Law.

4. Information Richness: Priority was given to cases where the reasoning section of the adjudicatory document was relatively detailed, clearly reflecting the court’s logic in determining compensation for mental distress in young children, the status of evidence admissibility, and whether the child’s specific circumstances were considered.

### 3.2. Data Collection

Judicial cases were retrieved from the Wolters Kluwer China Law & Reference Database, one of the most comprehensive and authoritative legal databases in China. The database provides access to officially published court judgments across all levels of the Chinese judiciary, including first-instance, appellate, and supreme court decisions. It is widely used by legal practitioners, scholars, and judicial institutions for research and professional reference. The database ensures the authenticity and reliability of case materials, as judgments are sourced from officially disclosed judicial documents.

The specific search criteria were set as follows: the document type was “Judgment”, the case type was “Civil”, the cause of action was “Dispute over Liability of Educational Institutions”, and the judgment date was “1 January 2017–31 December 2024.” The “Court’s Holding” section was required to contain terms related to “compensation for mental distress” or “solatium for mental distress”, and the “Facts Established” section was required to contain “kindergarten” or “preschool education institution”. The initial search yielded 351 judgments. Cases were excluded if they met any of the following criteria:

1. They Involved only adult plaintiffs or adult victims, even if the dispute arose from an educational institution.

2. They Mentioned children but the core legal dispute did not involve a claim for compensation for mental distress.

3. They Were duplicate judgments or rulings on the same case at different trial levels; these cases were excluded to avoid overrepresentation of a single dispute.

4. Judicial reasoning was overly simplistic or formulaic, resulting in an inability to effectively analyze the logic behind courts’ determinations of “seriousness.”

Ultimately, seven typical cases representing different types of disputes and adjudicative logics were selected. These seven cases are largely sufficient to cover the various adjudicative approaches existing in China’s current judicial practice, reflect the varying degrees of judicial attention paid to the specific circumstances of children, and provide a representative empirical basis for analyzing the current judicial dilemmas.

### 3.3. Data Analysis

For this study, we selected seven typical cases from recent years, covering areas such as unilateral dismissal of preschoolers by kindergartens (Case 1, Case 2), accidental injuries to preschoolers within kindergartens (Case 3, Case 4, Case 5), and preschoolers suffering from verbal abuse and corporal punishment by teachers (Case 6, Case 7). This study employed a qualitative multiple-case study, conducting a horizontal comparison across the seven cases in terms of their causes of action, plaintiffs’ claims, defendants’ arguments, courts’ findings of fact, adjudicative reasoning, and particularly the courts’ argumentation regarding the “severity” of mental distress, as well as the final judgments. By comparing these cases, the study sought to synthesize the core contradictions and regularities present in current judicial practice. The analysis focused on examining whether and how courts considered the physiological and psychological characteristics of young children across different case types, what evidence standards were adopted, and whether the adjudicative logic aligned with the principle of the Best Interests of the Child. The basic information and case summaries are presented in the table below.

## 4. Results

### 4.1. Analysis of Representative Cases and Adjudicative Approaches

Based on the above cases, this research identifies several adjudicative approaches in determining the severity of mental distress compensation for preschoolers.

In adjudicating compensation for mental distress, Chinese courts consistently emphasize the severity of the damage as a threshold requirement. For instance, in Case 1, the court underscored the “significant damage” to the child’s physical and mental health and the “irreversibility of early childhood education.” In Case 2, which also involved unilateral dismissal by a kindergarten, the court denied compensation due to insufficient evidence of severe consequences. In Case 5, the child’s death resulting from the kindergarten’s failure to fulfill its duty of care was deemed sufficient to satisfy the severity threshold. These cases demonstrate that severity functions as the decisive doctrinal filter in judicial reasoning.

However, judges lack a uniform adjudication standard for determining whether mental distress meets the severity requirement. Article 1183 of Chinese Civil Code strictly limits compensation to situations where “serious mental damage is caused.” Yet significant discrepancies appear in judicial application, mainly reflected in two determination pathways: “factual presumption” and “medical proof.” Crucially, these divergent approaches are not confined to a single court or region but are observable across different jurisdictions in China, from Beijing and Guangdong to Shaanxi and Heilongjiang, as illustrated in Table 1. This geographical and institutional diversity underscores that the inconsistency is a systemic issue within the Chinese judiciary, rather than an anomaly of a particular court.

The factual presumption model refers to situations in which courts infer the existence of severe mental distress directly from the nature of the wrongful act. Some courts, based on the psychological vulnerability of preschoolers, adopt the adjudication logic that “the act itself is the damage,” presuming the existence of severe mental distress without requiring additional proof of damage consequences. This model is particularly evident in cases involving unilateral dismissal of preschoolers by kindergartens and cases where children suffer verbal abuse or corporal punishment from teachers. Cases 1 and 7 exemplify this approach. In Case 6, although the cause of injury was unclear, the court similarly relied on children’s characteristics to support presumption.

By contrast, the medical proof model requires objective evidence, such as medical diagnoses, psychological assessment reports, or disability appraisals. In the absence of such documentation, claims for compensation are typically dismissed. This approach is especially prominent where psychological damage is relatively concealed or has not reached disability rating standards. For example, in Case 2 involving unilateral dismissal from kindergarten, the court stated that the existing evidence was insufficient to establish “serious mental distress” as defined by law. In Case 5, the court concluded that the plaintiff failed to prove sustained or long-term psychological harm. Case 3 similarly reflects reliance on objective evidentiary standards. This model emphasizes objective documentation and may not fully account for preschoolers’ cognitive and psychological expression abilities. Preschoolers often cannot accurately articulate mental suffering or cooperate with standardized psychological assessments. Moreover, China currently lacks assessment tools specifically designed for evaluating mental distress in preschoolers, and adult-centered appraisal standards may not adequately reflect their psychological condition.

The cases also reveal inconsistency in the consideration of preschoolers’ developmental characteristics. Some adjudications explicitly recognize children’s vulnerability and developmental particularities. For instance, Case 1 emphasized the irreversibility of early education, while Cases 6 and 7 acknowledged children’s emotional dependence and psychological fragility. However, many judgments only superficially acknowledge such particularities without substantive engagement, or entirely overlook the perspective of the preschoolers. Although Case 5 recognized children’s sensitivity to pain, it dismissed the claim due to lack of evidence of sustained abnormality. Similarly, in Case 2, the court characterized kindergarten transfer as causing certain mental pressure without further examination of its developmental implications. These variations suggest the absence of a consistent child-sensitive evaluative standard. Prevailing requirements such as “immediately visible,” “sustained existence,” and “medically confirmed” do not always align with the characteristics of early childhood psychological development.

### 4.2. Structural Causes of Adjudicative Inconsistency

Building upon the adjudicative approaches identified above, the observed adjudicative inconsistency in compensating preschoolers for mental distress can be explained from two structural dimensions: the normative level and the discretion level.

At the normative level, “severity,” as the core component of compensation for mental distress, constitutes an uncertain legal concept (Shu et al., 2020). Its interpretation requires both identification of objective harm and evaluative judgment regarding the extent of suffering. While this conceptual openness allows flexibility in judicial application, it also contributes to inconsistency in adjudication. Objectively, the severity of mental distress lacks quantifiable identification tools; subjectively, it highly depends on the judge’s personal value judgment on “what kind of pain deserves compensation,” significantly influenced by their life experience, knowledge structure, and understanding of child psychology, thereby leading to adjudication difference. The Civil Code and relevant judicial interpretations do not establish specific assessment criteria tailored to preschoolers, nor do they provide structured guidance for evaluating psychological harm in early childhood. As a result, courts apply a general standard that does not clearly differentiate between adult and child claimants.

At the discretion level, this normative indeterminacy expands judicial discretion. As observed in Section 4.1, some judges adopt a factual presumption approach, inferring severity from the wrongful act itself, particularly where children’s vulnerability is emphasized. Other judges adhere strictly to an adult-oriented medical proof model, requiring objective psychological or medical documentation and dismissing claims in which it is absent. The coexistence of these approaches reflects differing judicial interpretations of the severity requirement rather than uniform doctrinal application. It not only fails to follow the principle of “Best Interests of the Child” but also exacerbates inequities in the judicial protection of children’s rights (Zhong & Tang, 2022).

In summary, adjudicative inconsistency in mental distress compensation cases involving preschoolers arises from both the conceptual uncertainty of “severity” and the absence of child-specific normative guidance. The resulting variability in judicial discretion contributes to divergent adjudication outcomes and limits the consistent integration of preschoolers’ developmental particularities within existing legal standards.

## 5. Discussion

### 5.1. Interpreting Adjudicative Inconsistency in Light of Existing Scholarship

The findings of this research reveal that inconsistency in severity determination is not merely a matter of evidentiary variation but reflects deeper structural deficiencies in the legal framework governing compensation for preschoolers’ mental distress. Although courts uniformly recognize “severity” as the threshold requirement, they diverge between factual presumption and medical proof models. This divergence is amplified by the absence of child-specific evaluative criteria and by the open-textured nature of the concept of severity.

These findings resonate with existing scholarship on the marginalization of children’s voices in legal processes. Løvlie (2023), through empirical research on “voluntary assistance” cases in Norwegian child protection, notes that although preschoolers’ statements are procedurally documented, they are often marginalized and treated as “symbolic” rather than as “substantive evidence” integrated into decision-making. Similarly, the present research demonstrates that preschoolers’ developmental experiences are acknowledged rhetorically yet insufficiently integrated into severity assessments. The dominance of the medical proof model reflects an adult-centered evidentiary paradigm that prioritizes clinical documentation over developmental context.

At the same time, contemporary research challenges assumptions about preschoolers’ incapacity. Henderson-Dekort et al. (2023) contend that preschoolers are experts on their own lives in matters involving rights, participation, and capabilities. This position emphasizes the following: preschoolers have an irreplaceable direct cognitive authority over their own feelings, wishes, and experiences; with appropriate guidance through playing, drawing, and narrative, preschoolers can express complex concepts such as “rights”, “best interests”, and “capabilities.” Similarly, Mulvey et al. (2020) also demonstrate that preschoolers in conflict situations can evaluate behavioral responsibility through considerations like motivation, provocation, and intent, thereby challenging the viewpoint that their accounts are inadmissible. The inconsistency identified in this research therefore reflects not children’s inability, but institutional design failures (Fuentes & Vannelli, 2021). The principle of the Best Interests of the Child, though frequently invoked, lacks operational mechanisms in severity adjudication.

### 5.2. Operationalizing the Best Interests of the Child: The Dual-Model Framework

The findings of this research demonstrate that merely invoking the Best Interests of the Child principle does not resolve adjudicative inconsistency in determining severity. Without structured mechanisms, the principal risks remaining declaratory rather than operational. The central contribution of this research is therefore the development of a dual-model framework that integrates the Lundy model of participation with the Capabilities Approach for determining liability for compensation to preschoolers for mental distress, as shown in the figure below:

According to established legal doctrine, civil tort liability consists of four constitutive components: wrongful act, damage fact, causation, and subjective fault (L. Yang, 2013). The judicial controversy identified in this research primarily concerns the assessment of “damage,” specifically whether mental distress reaches the statutory threshold of severity. The dual-model framework does not alter the structure of tort liability but restructures how the element of damage is evaluated when the claimant is a preschooler.

Within this reconstructed understanding of the damage element, structured child participation becomes central to evidentiary assessment. Operationalized through the four interrelated dimensions of the Lundy model (space, voice, audience, and influence), participation is embedded directly into liability analysis. Space and voice create developmentally appropriate conditions for authentic expression, whereas audience and influence ensure that children’s views are directly considered and meaningfully incorporated into judicial reasoning, particularly in the assessment of severity and compensation. In this framework, preschoolers’ statements, whether documented through interviews, reports, or audiovisual materials, acquire evidentiary legitimacy alongside other statutory forms of proof. According to Article 66 of the Civil Procedure Law of the People’s Republic of China, revised in 2023, China’s civil litigation procedure includes seven statutory types of evidence. The preschoolers’ statement, formed in accordance with the Lundy model, along with other evidence constitutes part of the body of evidence in a case. Participation thus shifts from a symbolic procedural entitlement to a structured component of damage evaluation, enabling the Best Interests of the Child principle to function as an operational standard rather than a declaratory norm (Long & Grant, 2024).

**Figure 1:**
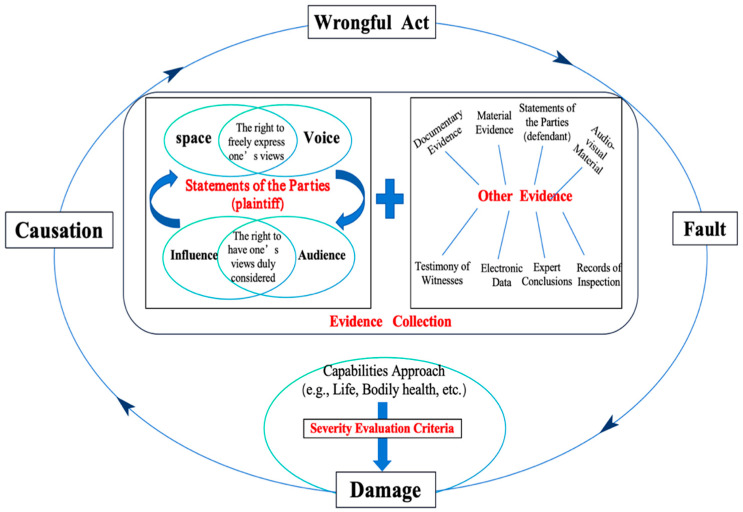
Dual-model framework for determining liability for mental distress in preschoolers.

In the stage of severity evaluation, as shown in Figure 1, the Capabilities Approach provides a multidimensional set of criteria for assessing damage within the structure of tort liability. Rather than limiting severity to clinically diagnosed psychological disorders, this approach evaluates whether the wrongful act has impaired core capabilities essential to early childhood development (Dixon & Nussbaum, 2011). Each core capability proposed by Nussbaum can correspond to specific behavioral manifestations or psychological reactions that may occur in preschoolers after damage, thereby providing a reference for evaluating “severity”. Moreover, because capabilities are shaped by individual characteristics and environmental conditions, severity assessment must be case-specific. The Best Interests of the Child principle therefore requires an individualized evaluation of damage, sensitive to each preschooler’s developmental context and vulnerabilities. Through this framework, the determination of damage moves from abstract statutory interpretation toward a structured, child-centered assessment aligned with developmental realities.

### 5.3. Judicial and Institutional Implications

The findings of this study and the proposed dual-model framework carry broader implications for judicial reasoning and the institutional development of children’s rights within civil liability systems. Current severity determinations in mental distress cases involving preschoolers remain largely dependent on adult-centered evidentiary standards. Such standards risk overlooking developmental trauma, whose effects may be latent, cumulative, and expressed through behavioral or relational disruption rather than formal diagnosis (Thoma et al., 2021). When adjudication fails to incorporate these developmental realities, the Best Interests of the Child principal risks remaining rhetorical rather than operational. The theoretical significance of this study lies in its integration of child participation theories and the Capabilities Approach into the civil tort liability system, thereby expanding the analytical toolkit for children’s rights protection. By reframing severity in terms of capability impairment and structured participation, the dual-model framework offers a principled method for reducing discretionary inconsistency and reinforcing doctrinal coherence. In this way, the framework strengthens the analytical integrity of tort doctrine while aligning liability standards with contemporary children’s rights theory (Z. Yang, 2022).

Institutionally, the implications of this research extend beyond individual cases. Recent developments in Chinese judicial practice suggest a growing recognition of the distinctive needs of child victims, including interpretive flexibility in awarding mental distress compensation within criminal incidental proceedings. However, such developments remain fragmented and lack a unified analytical structure. The dual-model framework offers a replicable methodology through which courts may systematically integrate participation and developmental evaluation into severity determination, even in the absence of detailed legislative standards. Its practical implications are substantial, offering Chinese courts a developmentally appropriate and operable judicial discretion framework following the implementation of the Preschool Education Law. In doing so, it facilitates a shift in mental distress compensation for children from “formal protection” towards “substantive justice.”

## 6. Limitations and Future Research Directions

While these findings advance our understanding of preschoolers’ rights and the judicial assessment of mental distress, several limitations should be acknowledged. First, the research relies on an interdisciplinary perspective and analysis of typical cases to examine the judicial status quo and construct a severity standard model for compensation to preschoolers for mental distress. While this approach supports a normative and contextual understanding, it does not employ quantitative or longitudinal methods to operationalize the “severity” standard or track psychological and behavioral development over time. Future studies could incorporate empirical designs, such as psychological scales or longitudinal studies, to test the model’s applicability and robustness in judicial settings. Second, the sample of publicly available cases on compensation to preschoolers for mental distress is limited and predominantly involves specific disputes, such as dismissals and institutional liability. Future studies should expand case collection to include more varied samples to enhance the generalizability of the proposed model. Finally, due to the sensitivity of minor-related information, judicial practice often anonymizes and simplifies case details, particularly concerning psychological status, verbal expressions, and family background, to protect privacy and well-being. These constraints limit in-depth case analysis and nuanced assessment of children’s subjective experiences in severity determination. Future work could, under strict ethical approval and authorized collaboration with judicial or child protection agencies, use non-identifiable data or case studies to better understand how children’s voices are heard and integrated in legal processes.

## 7. Conclusions

This research examined the judicial determination of “severity” in mental distress compensation cases involving preschoolers in China and identified significant inconsistency rooted in adult-centered evidentiary standards and the indeterminate interpretation of damage. Through a qualitative multiple-case analysis, the findings reveal that current adjudication oscillates between factual presumption and medical proof models, often neglecting the developmental particularities and participatory rights of preschool children. As a result, the Best Interests of the Child principle risks functioning as a rhetorical reference rather than a substantive evaluative standard.

To address this structural gap, this research proposed a dual-model framework integrating the Lundy model of participation and the Capabilities Approach. By embedding structured child participation into evidentiary processes and introducing developmental criteria into severity assessment, the framework reconstructs the “damage” element within tort liability doctrine. Rather than creating a separate liability regime for children, it refines existing legal structures to better reflect early childhood vulnerability and capability deprivation. In doing so, the framework transforms the Best Interests of the Child principle from an abstract normative guideline into an operational judicial methodology.

This research’s implications extend beyond individual adjudication. Comparative developments in international children’s rights jurisprudence show that courts can invoke participatory and developmental principles to interpret open-ended legal standards and strengthen the protection of children’s rights, even in the absence of detailed legislative guidance. In this context, the proposed framework offers a structured and replicable approach for integrating child-centered reasoning into domestic liability analysis. By aligning tort doctrine with developmental realities and participatory rights, it contributes to the gradual institutionalization of children’s rights within civil adjudication.

## Figures and Tables

**Table 1:** Typical cases of compensation to preschoolers for mental distress in China.

	Case Number	Case Name	Brief Case Summary	Summary of the Reasoning of the Court	Whether the Case Supports Compensation for Mental Distress	Consideration of Preschoolers’ Particularities in Adjudication
1	(2022) Guangdong 06 Civil Final No. 15098	Ye vs. A Kindergarten Education Training Contract Dispute Case	The defendant kindergarten expelled the child following the plaintiff’s mother’s complaint about its alleged irregularities, thereby shifting the dispute between the parents and the kindergarten onto the child.	The kindergarten dismissed the child following conflicts with the parents, leading to physical and psychological harm. Considering the specific circumstances, the court awarded the child ¥10,000 in compensation, while rejecting the plaintiff’s claim for a higher amount. It should be noted that early education is irreversible.	√	√
2	(2024) Shanxi 01 Civil Final No. 19263	Civil judgment of the second instance on the mental distress compensation dispute between Wang and kindergarten in Xi’an New City	After noticing scratches on her child Liu’s face and receiving no satisfactory response from the kindergarten, the mother reported the incident to the police. The kindergarten expelled the child and issued a refund.	Compensation for mental distress requires proof of severe psychological harm in accordance with legal standards. In this case, although the child experienced mental pressure after discontinuing attendance at the kindergarten, the evidence was insufficient to establish “severe mental distress” as defined by law.	☓	☓
3	(2024) Beijing 0105 Civil First No. 36804	Civil judgement of the first instance of the dispute over the liability of an educational institution between Li and a Beijing company	During an activity at the defendant’s park, the plaintiff’s child climbed onto a platform adjacent to the ocean ball pool and fell, sustaining a fracture.	The plaintiff sought compensation for mental distress, claiming the accident severely affected him and his family, but submitted no supporting evidence. Based on the infringement, consequences, fault degree, and other relevant factors, the court awarded ¥5000 as compensation. As a children’s care institution, the defendant must fulfill its duty of care in accordance with the developmental needs of preschoolers and safeguard their physical and mental health as well as their legal rights.	√	√
4	(2024) Liaoning 07Civil Final No. 2682	Second-instance judgement in the dispute between Miao and a kindergarten over the liability of an educational institution	The appellant sustained injuries during a physical education class at the appellee’s kindergarten due to inadequate safety measures and the appellant’s status as a minor lacking full capacity.	The court dismissed the appellant’s claim for mental distress due to the absence of a disability assessment and lack of substantiating evidence.	☓	☓
5	(2024) Guangdong 0705 Civil First No. 150	Civil judgement of first instance in a dispute over the liability of B educational institution	A daycare provider was unable to demonstrate that a child’s injury either occurred prior to enrollment or was self-inflicted while under its supervision.	Children possess limited emotional regulation and heightened pain sensitivity, making crying a normal response to injury. The plaintiff failed to demonstrate that the incident resulted in sustained physiological or psychological abnormalities or permanent harm to the child, nor evidence of severe mental distress.	☓	√
6	(2018) Heilongjiang 10 Civil Final No. 37	Civil judgement in the dispute between a kindergarten and Song on the liability of an educational institution	The child sustained head and facial injuries while attending the defendant’s kindergarten. The defendant alleged the injury was accidental, whereas the plaintiff accused the teacher of physical abuse; however, neither party provided substantiating evidence regarding the cause.	Given the plaintiff’s young age and psychological vulnerability, the court recognized that damage occurring in an educational setting could cause lasting trauma and mental distress. Accordingly, the claim for mental distress was upheld. Considering the degree of fault, damage, and local living standards, the court awarded ¥4000 for compensation.	√	√
7	(2017) Shanxi 01 Civil Final No. 2081	Civil judgment of the second instance on the tort liability dispute between Zhang and the kindergarten teacher	At the age of two and a half, the appellee was enrolled in daycare, where a teacher engaged in inappropriate restraint of young children through verbal abuse and physical acts such as pressing a shoe against the child’s forehead. This finding is substantiated by evidence including audiovisual recordings, statements from both parties, and the ruling of the Board of Education.	Although the child’s injuries lacked clear medical diagnosis, they constitute an objective fact. The teacher, who served as a primary attachment figure for the child, perpetrated verbal and physical abuse that exceeded the child’s capacity to endure. The severity of damage in this case can be reasonably inferred by a reasonable person without further evidence. The Court accordingly dismissed the appellant’s claim.	√	√

## Data Availability

The data presented in this study are openly available from Wolters Kluwer at https://law.wkinfo.com.cn, accessed on 1 June 2025.
